# Anomalous vertebral arteries and recurrent ischemic stroke: Insights from a unique case (first documented case-report from Syria)

**DOI:** 10.1097/MD.0000000000043166

**Published:** 2025-07-04

**Authors:** Mohamad H. Mosi, Nawwar Soliman, Moaaz Khalouf Alshaar, Ghassan Hamzeh

**Affiliations:** a Department of Internal Medicine, Al Assad and Al Mouwasat University Hospitals, Damascus University Faculty of Medicine, Damascus, Syria; b Hama University Faculty of Medicine, Hama, Syria; c Neurology Department, Al Assad University Hospitals, Damascus University Faculty of Medicine, Damascus, Syria.

**Keywords:** Circle of Willis, diabetic ketoacidosis, inspiration pneumonia, ischemic stroke, posterior circulation, turbulent blood flow

## Abstract

**Rationale:**

The posterior circulation represented by the vertebrobasilar artery system is responsible for 20% of brain perfusion. Posterior circulation ischemia can cause a variety of symptoms due to the broad area supplied by the vertebrobasilar artery. In addition, stroke is the second leading cause of death globally, highlighting the importance of studying any potential contributing factors.

**Patient concerns:**

A 68-year-old nonsmoker and nonalcoholic with a history of diabetes type 2 and a previous stroke, presented with 2 days of visual disturbance with a burning sensation associated with occipital headache, fever, and polyuria.

**Diagnosis:**

He was admitted to our hospital due to his symptoms and during his evaluation, a computed tomography scan was performed and showed a bilateral calcification on the vertebral arteries. Magnetic resonance angiography showed a variant vessel connected between the vertebral arteries at the level of medulla.

**Intervention:**

The patient was admitted to the intensive care unit after being diagnosed with stroke complicated with aspiration pneumonia and diabetic ketoacidosis. He was treated with IV fluids and insulin pump without using thrombolysis or vascular intervention due to the time of symptoms.

**Outcomes:**

The patient’s condition and laboratory tests improved and was discharged after 2 weeks.

**Lessons:**

This study investigates an extremely rare variant of the Circle of Willis and the potential consequences associated with such a variant, including stroke, which is considered one of the most lethal outcomes. The study highlights the importance of investigating the association between this variant and the incidence and frequency of stroke. The patient in the study developed stroke twice and atherosclerosis due to this variant.

## 1. Introduction

The Circle of Willis (CoW) is formed initially by 2 major pairs of arteries: the right and left internal carotid arteries, which originate from the common carotid arteries (the left from the aorta and the right from the brachiocephalic trunk), and the right and left vertebral arteries, both of which arise from the subclavian arteries. These arteries play a fundamental role in shaping the CoW. This type of formation creates an arterial network that supports the brain by providing perfusion and collateral circulation, particularly during hypoperfusion states.^[1]^ The CoW comprises arteries from both the anterior and posterior circulations. The anterior circulation includes the internal carotid arteries, anterior cerebral arteries, anterior communicating artery, and middle cerebral arteries, while the posterior circulation consists of the vertebral arteries, basilar artery, posterior cerebral arteries, and posterior communicating arteries.^[2]^

CoW variations are most common in the anterior circulation compared to the posterior.^[3]^ Some studies have shown an association between variations and some neuro-diseases like: migraine,^[4]^ ischemic stroke,^[5]^ intracranial aneurysm formation.^[6]^

Stroke is the second cause of death globally and the fifth cause in the USA.^[5]^ Ischemic stroke can be classified into 3 main subtypes: thrombosis, embolism, and systemic hypoperfusion.^[7]^ In 2020, 7.86 million people suffered from ischemic strokes, and 3.15 million of those cases resulted in death. Furthermore, it is projected that the incidence of ischemic strokes will rise to 9.62 million by 2030.^[8]^

The importance of CoW variations is reflected by the diseases that may be associated with those variations. In addition, knowing and studying the variations play an important role for the neuro and vascular surgeon during vasculo-neuro procedures.

In this study, we are exploring the relationship between an extremely rare variant – a connection between the V4 segments of the 2 vertebral arteries – and the incidence of strokes in the posterior circulation.

## 2. Case report

A 55-year-old nonsmoker and nonalcoholic male was admitted to emergency room after 2 days of visual disturbance in the left eye with a burning sensation in the left hemi-body associated with occipital headache. In addition, he suffered from polyuria, severe dry mouth, vomiting, productive yellow cough, fever, and abdominal pain.

**Figure 1.**
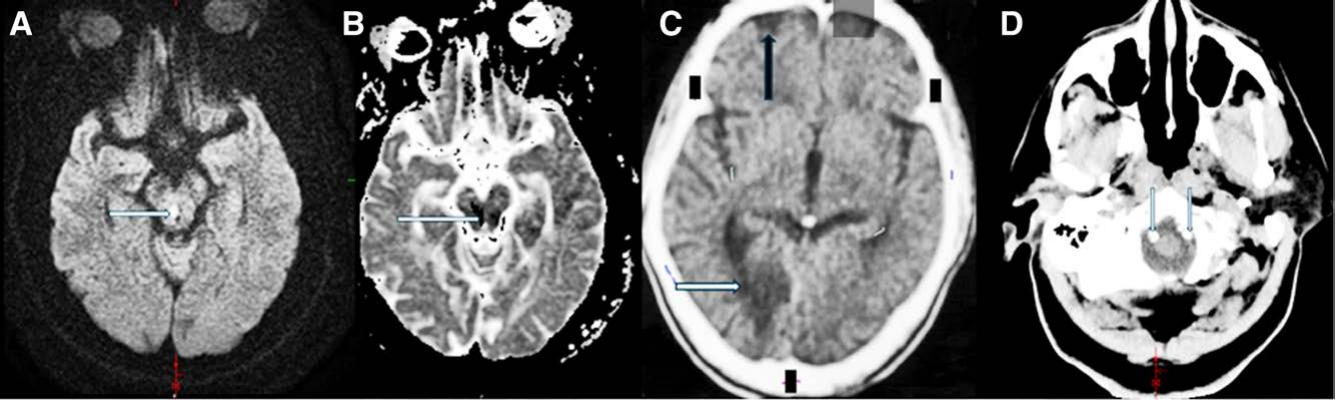
The images (A and B above) show high signal in mid-brain area next to cerebral aqueduct (b1000 A image, long white arrow) and low signal in same area (A–D image, long white arrow), reflecting infarction. CT images (C and D) reveal calcifications on both vertebral arteries (2 white arrows on D image) and sulci widening with infarction on posterior cerebral artery territory (C image black and white, respectively). CT = computed tomography.

The patient has a history of type 2 diabetes mellitus, chronic vertigo, and ischemic stroke with a previous admission 6 years ago with left-side weakness (mid-brain infarction, Fig. 1A and B).

On admission, the patient was presented with a fever and was in severe condition. His vital signs were as follows: temperature of 38.0°C, blood pressure of 121/74 mm Hg, pulse rate of 110 beats per minute (regular), respiratory rate of 25 breaths per minute, and oxygen saturation of 70% on room air. The patient’s body mass index was 34.39.

Physical examination showed that the patient was confused with a fruity smell from the mouth and coarse crackles heard at the base of both lungs. The neurologic examination revealed that the Glasgow Coma Scale (GCS) was 14/15 with upper and lower limb weakness and diplopia, left homonymous hemianopsia and the rest of the examination was unremarkable (the pupils were symmetrical, equal in size, and reactive to light; reflexes in the right side were +3 and +1 in left side, meningeal signs and Babinski were negative).

**Figure 2.**
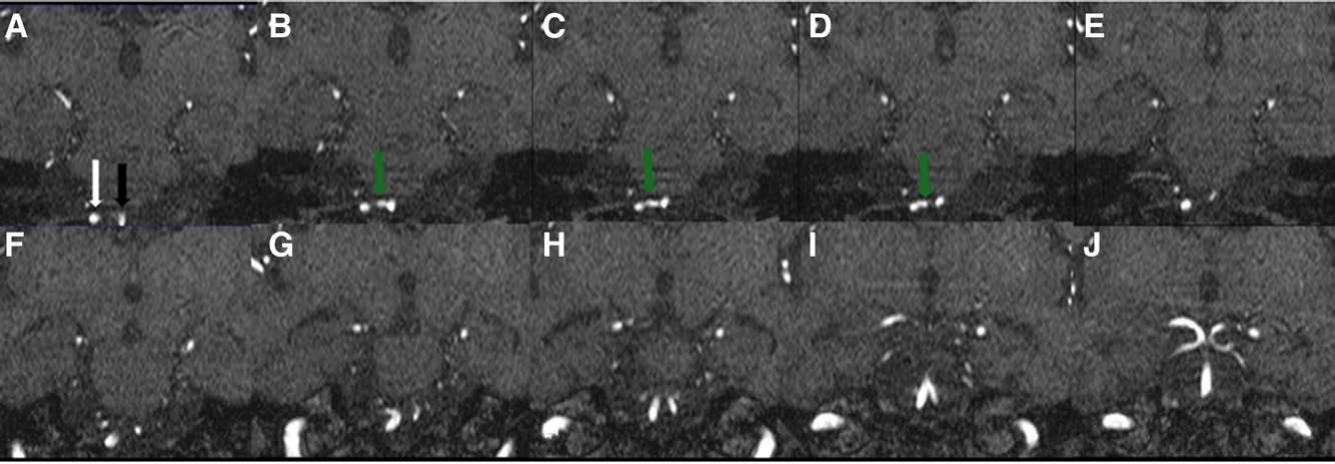
Coronal MRA slides reveal right and left vertebral arteries (white and black arrows) with the arterial variant that connects right and left vertebral arteries (green arrow). MRA = magnetic resonance angiography.

**Figure 3.**
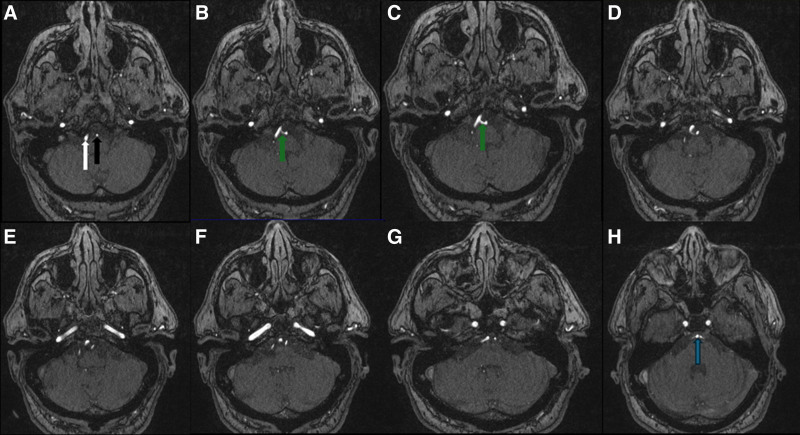
Axial MRA slides from A to H reveal right and left vertebral arteries (white and black arrows, slide A) with the arterial variant that connects right and left vertebral arteries (green arrow). Basilar artery (blue arrow). MRA = magnetic resonance angiography.

**Figure 4.**
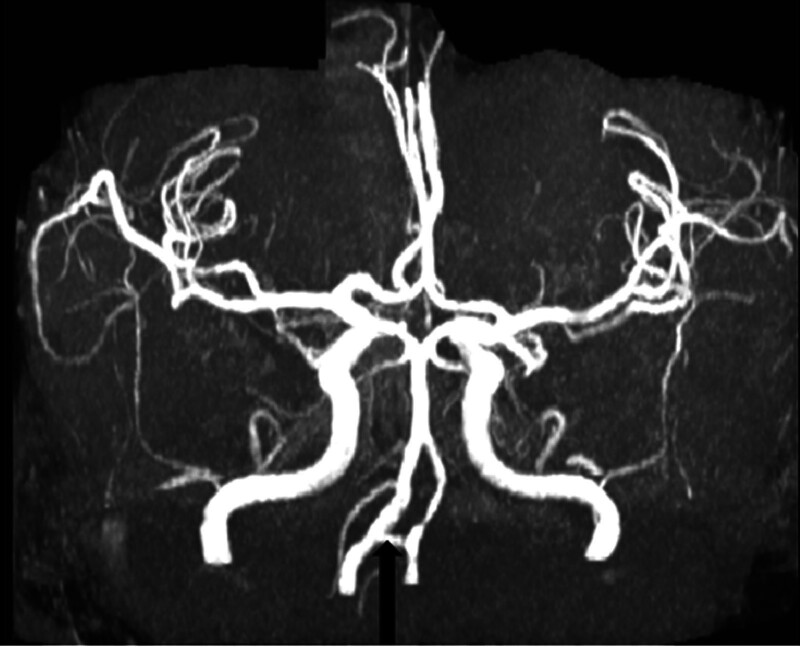
3D reconstruction of MRA shows the variant in Circle of Wills (the artery that connects vertebral arteries), long black arrow. MRA = magnetic resonance angiography.

The initial laboratory tests are shown in Table 1. Electrocardiogram showed sinus tachycardia. Chest X-ray revealed mild basilar right opacities, reflecting aspiration pneumonia. A brain computed tomography without contrast scan at the time of admission showed global sulci widening with infarctions and foci in the right posterior and middle cerebral arteries (temporal and occipital lobes) with bilateral calcification on both vertebral arteries (Fig. 1C and D). A magnetic resonance angiography showed stenosis on the right posterior cerebral artery with an artery connecting both vertebral arteries at the level of medulla (Figs. 2–4). The patient’s laboratory examination, tests, and radiologic images corresponded with ischemic stroke and aspiration pneumonia associated with diabetic ketoacidosis.

**Table 1:** Initial laboratory tests.

WBC	12 (H)	Na	137	CRP	20
L/N	19/80	K	3.7	PTT	38
Hb	12.6	Cl	105	INR	1.7
HT	40	Ca	8.5	ABGs analyses	
Plt	192	Glu	400	PH	7.30
Cr	2	HbA1	10.3%	PCO2	20
urea	40	ALT	55	PO2	48
GFR	50	AST	47	HCO3	12
TG	100	LDL	106	AG	20
Cholesterol	170	HDL	40		

ABG = atrial blood gases, AG = anion gap, ALT = alanine transaminase, AST = aspartate transaminase, Ca = calcium, Cl = chloride, Cr = creatinine, CRP = C-reactive protein, GFR = granular filtrating rate, GLU = glucose, Hb = hemoglobin, HbA1 = haemoglobin A1, HDL = high density lipoprotein, HT = hematocrit, INR = international normalized ratio, K = potassium, L = lymphocytes, LDL = low density lipoprotein, N = neutrophils, Na = sodium, Plt = platelets, PTT = prothrombin time, TG = triglycerides, WBC = white blood cells.

The patient was admitted to the intensive care unit and started on a ventilator mask, aspirin 81 mg once daily, atorvastatin 40 mg once daily, insulin pump 1 unit/kg/h, intravenous IV saline, IV potassium chloride, subcutaneous (SC) heparin, ceftriaxone 1 g/12 h and metronidazole 500 mg/8 hr with monitoring for electrolytes, glucose, arterial blood gas, vital signs, and GCS.

Tissue plasminogen activator and vascular intervention were not available due to symptoms time. The microbial culture was positive for Klebsiella sensitive to ceftriaxone (metronidazole was stopped). Two days after admission, the patient’s condition improved, and he was able to eat. IV insulin was stopped with starting SC insulin (bolus and basal) and physiotherapy. During the time in the intensive care unit, the patient experienced a one-time seizure and was treated with phenytoin 15 mg/kg IV with maintenance 100 mg/8 h IV. In addition, the fever improved and nasal cannula was started with improvement in GCS to 15/15.

After 2 weeks of admission, the patient’s status and blood tests improved, as shown in Table 2, and he was discharged on the following medications (oral phenytoin 100 mg/8 h, bolus basal SC insulin, rivaroxaban 2.5 mg twice, aspirin 81, and atorvastatin 40 mg once daily).

**Table 2:** Laboratory tests on discharge.

WBC	4.4	Glu	120
L/N	25/70	CRP	0.5
Hb	12.2	ABGs analyses	
Plt	237	PH	7.42
Na	136	PCO2	35
K	4	PO2	65
Cl	102	HCO3	24
Cr	2.1	AG	10

ABG = atrial blood gases, AG = anion gap, Cl = chloride, Cr = creatinine, CRP = C-reactive protein, Glu = glucose, K = potassium, L = lymphocytes, N = neutrophils, Na = sodium, Plt = platelets, WBC = white blood cells.

## 3. Discussion

Stroke prediction and analyzing its risk factors are an important aspect leading to the improvement of patient prognosis. Vascular risk factors and stroke incidence were investigated, finding that stroke (ischemic and hemorrhage) is more common in males compared to females. In addition, it was found that hypertension is the most important contributor to stroke compared to other factors. Other key factors include diabetes, diet, smoking, and alcoholism.^[9]^ Our patient has a history of uncontrolled diabetes mellitus type 2 which is considered a risk factor for stroke alongside with aspiration pneumonia which is a result of swallowing reflex absence due to stroke. Such situations cause stress status and increase blood glucose levels and the incidence of stroke. The increased level of fasting blood glucose was analyzed as a risk factor for having first-time stroke in patients, finding in Yu-qing Huang et al^[10]^ that high level of fast blood glucose is associated with risk for having first-time stroke. However, our patient has uncontrolled diabetes and already had >1 episode of stroke. In such a scenario, studying this association is essential.

Identifying CoW as normal without variants depends on 4 criteria, including the presence of all arterial segments with absence of any additional segments, measuring >1 mm diameter, and rising from their natural origin.^[11]^ However, CoW has a higher probability of having at least 1 variant compared to a normal completed 1 (45.2% of 1000 autopsies had complete normal CoW^[12]^). Such variants include duplication or agenesis of one of the segments, fenestration, and absence of vessels in some locations. Fenestration is reflected by separation of 1 segment to 2 parts then combining again in the same arterial segments. In addition, duplication means that single segments become doubled from their origin to their ends.^[13]^ Our patient has an extremely rare variant of vertebral arteries which leads to hemodynamic changes and turbulent blood flow. This finding collaborates with the synergistic effect of other stroke risk factors, such as poorly controlled type 2 diabetes mellitus, in increasing the incidence of atherosclerosis, plaque formation, and recurrent stroke—particularly within the posterior circulation. Imaging revealed atherosclerotic plaques involving both vertebral arteries, which may explain the occurrence of multiple strokes in this vascular territory (Fig. 1). Nevertheless, many studies have been investigating the relationship between blood flow, CoW’s branches and variants such as Rovshan M Ismailov^[14]^ and yet there is no definitive explanation for atherosclerosis formation in such a scenario and more studies need to be conducted. Others investigated the correlation between carotid atherosclerosis (plaques) and CoW with variations, indicating that patients with CoW variations have more likelihood to have plaques and stenosis in the carotids.^[15]^ The collaboration between CoW variants and the changes in hemodynamic status and blood flow have been studied,^[16]^ however, some studies such as Lin et al^[12]^ were conducted to investigate the association between incomplete CoW and stroke, which concluded that there is a decrease in the odds of having good outcome stroke by 47% in CoW with variant or incomplete with correcting to sex and age. Moreover, other studies such as Westphal et al^[17]^ could not find that association, leaving this scenario controversial. Soares et al^[18]^ investigated the relationship between postoperative stroke occurrence and the anatomical completeness of CoW. The study cohort included 54 patients with a complete CoW and 44 patients with an incomplete CoW. The incidence of stroke was reported at 3.1%, with no statistically significant difference observed between the 2 cohorts (*P* = .68). The findings underscore the need for further investigations involving larger cohorts to better explore potential relationships between CoW completeness and stroke risk. Vertebrobasilar insufficiency identifies insufficient blood flow through the posterior circulation of the brain, which is formed by the vertebral arteries and their combing artery (basilar). The vertebrobasilar system perfuses areas including the brainstem, thalamus, hippocampus, cerebellum, occipital, and medial temporal lobes. These broad areas are responsible for the variety of symptoms that can occur during posterior circulation ischemia (PCI), such as upper-lower limb weakness, headache, change in level of consciousness, hemiparesis and homonymous hemianopsia.^[19]^ Our patient had a history of a previous stroke complicated with persistent left-side weakness and vertigo and a diagnosis ofPCI (in the midbrain) and now his new symptoms of headache and visual disturbance are correlated with a recurrent PCI, confirmed by the computed tomography imaging during his admission (Fig. 1). These recurrent stroke episodes (both episodes in the posterior circulation which is less common to have stroke compared to anterior) are explained by the synergetic effects of persistent risk factors like DM type 2 and the hemodynamic changes caused by the variants of vertebral arteries.

## 4. Conclusion

This study investigates an extremely rare variant of the CoW and the potential consequences associated with such a variant, including stroke, which is considered one of the most lethal outcomes. The study highlights the importance of investigating the association between this variant and the incidence and frequency of stroke. The patient in the study developed stroke twice and atherosclerosis due to the synergetic effect of uncontrolled diabetes and this variant.

## Acknowledgments

The authors thank the patient for his approval to publish this case report.

## Author contributions

**Data curation:** Mohamad H. Mosi, Nawwar Soliman.

**Investigation:** Moaaz Khalouf Alshaar.

**Resources:** Mohamad H. Mosi, Moaaz Khalouf Alshaar.

**Supervision:** Ghassan Hamzeh.

**Writing – original draft:** Mohamad H. Mosi, Nawwar Soliman.

**Writing – review & editing:** Nawwar Soliman.
